# Opportunities for Understanding MS Mechanisms and Progression With MRI Using Large-Scale Data Sharing and Artificial Intelligence

**DOI:** 10.1212/WNL.0000000000012884

**Published:** 2021-11-23

**Authors:** Hugo Vrenken, Mark Jenkinson, Dzung L. Pham, Charles R.G. Guttmann, Deborah Pareto, Michel Paardekooper, Alexandra de Sitter, Maria A. Rocca, Viktor Wottschel, M. Jorge Cardoso, Frederik Barkhof, on behalf of the MAGNIMS Study Group

**Affiliations:** From the MS Center Amsterdam (H.V., A.d.S., V.W.), Amsterdam Neuroscience, Department of Radiology and Nuclear Medicine, Amsterdam UMC (M.P.), the Netherlands; Wellcome Centre for Integrative Neuroimaging (WIN), FMRIB (M.J.), Nuffield Department of Clinical Neurosciences (NDCN), University of Oxford, UK; Human Imaging and Image Processing Core (D.L.P.), Center for Neuroscience and Regenerative Medicine, The Henry M. Jackson Foundation, Bethesda, MD; Center for Neurological Imaging (C.R.G.G.), Department of Radiology, Brigham and Women's Hospital, Boston, MA; Section of Neuroradiology (Department of Radiology) (D.P.), Vall d’Hebron University Hospital and Research Institute (VHIR), Autonomous University Barcelona, Spain; Neuroimaging Research Unit (M.A.R.), Institute of Experimental Neurology, Division of Neuroscience, IRCCS San Raffaele Scientific Institute, Milan, Italy; AMIGO (M.J.C.), School of Biomedical Engineering and Imaging Sciences, King's College London; and Institutes of Neurology & Healthcare Engineering (F.B.), UCL London, UK.

## Abstract

Patients with multiple sclerosis (MS) have heterogeneous clinical presentations, symptoms, and progression over time, making MS difficult to assess and comprehend in vivo. The combination of large-scale data sharing and artificial intelligence creates new opportunities for monitoring and understanding MS using MRI. First, development of validated MS-specific image analysis methods can be boosted by verified reference, test, and benchmark imaging data. Using detailed expert annotations, artificial intelligence algorithms can be trained on such MS-specific data. Second, understanding disease processes could be greatly advanced through shared data of large MS cohorts with clinical, demographic, and treatment information. Relevant patterns in such data that may be imperceptible to a human observer could be detected through artificial intelligence techniques. This applies from image analysis (lesions, atrophy, or functional network changes) to large multidomain datasets (imaging, cognition, clinical disability, genetics). After reviewing data sharing and artificial intelligence, we highlight 3 areas that offer strong opportunities for making advances in the next few years: crowdsourcing, personal data protection, and organized analysis challenges. Difficulties as well as specific recommendations to overcome them are discussed, in order to best leverage data sharing and artificial intelligence to improve image analysis, imaging, and the understanding of MS.

Multiple sclerosis (MS) is highly heterogeneous across patients in terms of symptoms, sites of damage, degree of recovery, and development of the disease across time. Relevant patterns may be imperceptible to a human observer, but by analyzing large amounts of imaging data with sophisticated artificial intelligence techniques, urgently required advances in understanding MS disease pathologic heterogeneity may be made.

Furthermore, to track disease progression in individual patients with MS, MRI markers are needed. This could benefit from MS-specific image analysis methods, because existing generalized methods tend to exhibit poorer performance in patients with MS,^1^ as has been demonstrated quantitatively for segmentation of deep gray matter structures.^2^ Large amounts of MS imaging data, with expert annotations as appropriate, can be used to train and validate more accurate measurement and analysis tools specifically for MS.

Against this background, we review the possibilities of data sharing and artificial intelligence for improved applications of MRI to study MS, addressing both the need to understand MS disease processes and the need for MS-dedicated quantitative measurement and analysis techniques for MRI assessments. We first survey relevant existing efforts regarding data sharing and artificial intelligence, and then highlight 3 areas of interest in bringing the field forward: crowdsourcing, personal data protection, and organized analysis challenges (see the Figure for the methods used to create this article). Specific recommendations aim to achieve the best outcomes for patients with MS.

**Figure:**
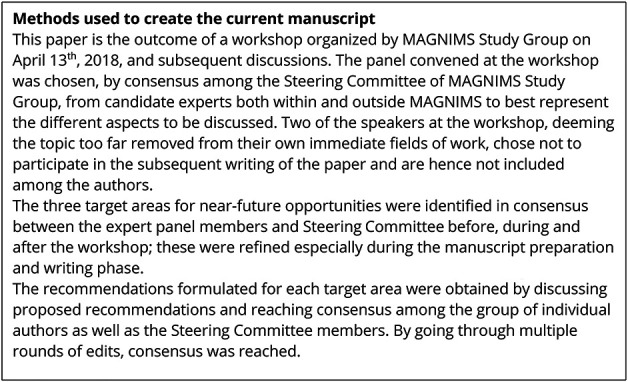
Methods Used to Create the Article

## Data Sharing

### Data Sharing in MS: Nonimaging

The multicenter collection of MS clinical data provides valuable information on disease prevalence, current treatment patterns, and general distribution of patients' outcomes. Thus, clinical registries including data from several MS centers have been strongly promoted in the past decades. National and regional MS registries exist in most countries, especially in Europe^3^ and North America.^4^ Data collected by national initiatives have often been included in computerized platforms, such as the European Register for Multiple Sclerosis (EUReMS)^5^ or the MSBase.^6^ Collaborative research studies have used these data to define the value of prognostic indicators in different patient populations,^7^ to investigate the influence of demographic and geographic factors on MS clinical course,^8^ and to evaluate the comparative efficacy of different drugs^9^ (additional references in eAppendix 1, https://doi.org/10.5061/dryad.2fqz612p9).

### Data Sharing in Neuro-MRI: Non-MS

Data sharing in MRI is becoming increasingly prevalent, large-scale, and open in the neuroimaging community. Table 1 lists several prominent examples, including the Alzheimer's Disease Neuroimaging Initiative (ADNI), which in the neuroimaging field is a template for data acquisition and fostering of methodologic developments. These datasets are associated with a range of access policies (from free, unrestricted downloads to collaboration-only agreements) and cover various sizes, demographics, and pathologies. They provide access to large, diverse groups of subjects, including rare diseases, and a wider range of disease stages (including prodromal cases) than is possible from single studies. In addition, the increasingly large numbers provide greater statistical power and the opportunity to apply state-of-the-art deep learning techniques. They also allow common standards to be applied in the evaluation of methodologic tools, as pioneered by the MICCAI challenges.^e67^ As such, they provide the community with fair and open comparisons of methods, a richer set of data on which to test hypotheses, and greater ability for assessing reliability and repeatability. There are also benefits for those involved in creating and managing such datasets, because the process of designing, piloting, and preprocessing provides impetus for novel developments in acquisition and analysis, demonstrated by state-of-the-art methodologies developed within the Human Connectome Project. In addition, there are benefits in visibility, engagement, and publications. Challenges still exist (e.g., information technology infrastructure, access policies, ethics policies) but many such datasets are already accessible, with a range of solutions to these problems, thus offering options for the creation of new datasets focusing on MS. The datasets listed also highlight that standardized magnetic resonance acquisition protocols can harmonize data only to a certain extent. Therefore, alternative approaches such as synthetic MRI should also be investigated (additional references in eAppendix 1).

**Table 1:**
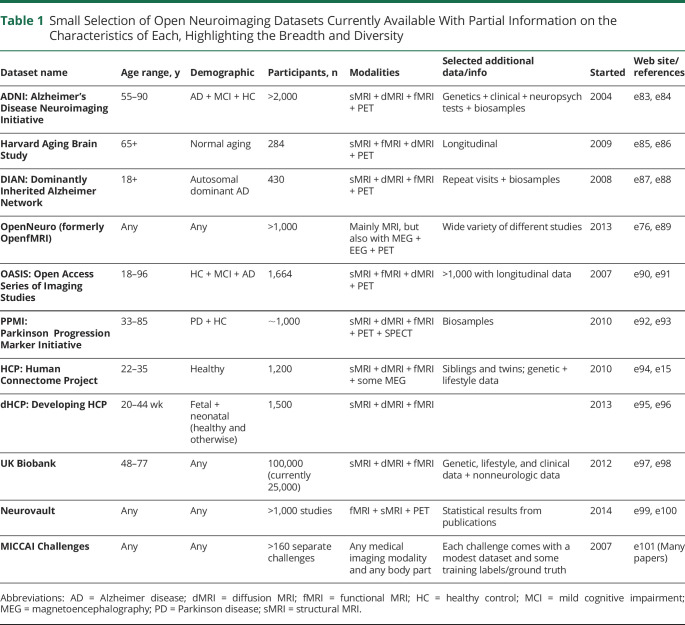
Small Selection of Open Neuroimaging Datasets Currently Available With Partial Information on the Characteristics of Each, Highlighting the Breadth and Diversity

### Data Sharing of MRI in MS

MRI is one of the most important tools for diagnosing and monitoring MS.^10^ However, MRI data collected by clinical MS registries usually include only conventional measures or metadata regarding fulfilment of diagnostic criteria.^5,6^ Recent collaborations (for example, the publicly funded German Competence Network Multiple Sclerosis^e68^ or the privately funded MS PATHS^11^) promoted the use of relatively standard conventional MRI protocols (typical T1-weighted, proton density–weighted, and T2-weighted or fluid-attenuated inversion recovery images), but did not include advanced MRI techniques (such as quantitative mapping techniques of tissue properties, tractography, spectroscopy, or functional MRI). Table 2 lists MS Registries identified from public sources that collect MRI information.

**Table 2:**
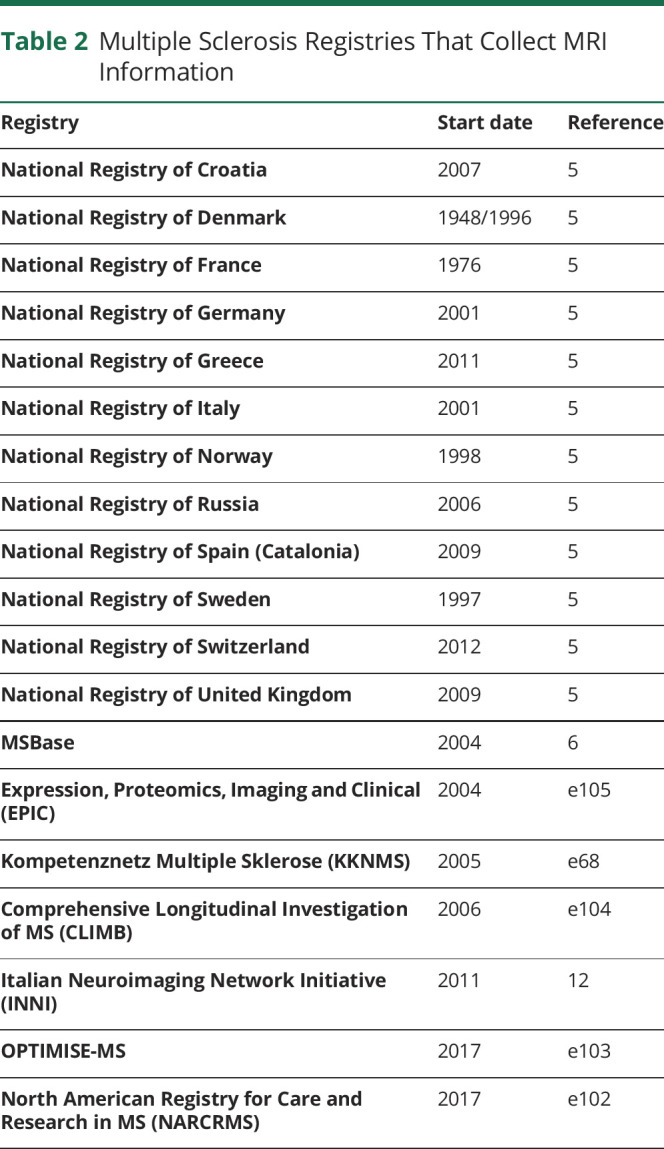
Multiple Sclerosis Registries That Collect MRI Information

As an example, the Italian Neuroimaging Network Initiative (INNI)^12,e69^ has recently been established among 4 sites leading MRI research in MS in Italy, with the support of the Italian MS Society. INNI's major goal is to determine and validate novel MRI biomarkers, including biomarkers based on more advanced, nonconventional imaging techniques, to be utilized as predictors or outcomes in future MS studies. INNI aims also to standardize MRI procedures of acquisition and analysis in MS at a national level.

A large population of patients with MS and healthy controls (more than 1,800 participants and more than 3,000 MRI examinations) has been collected in the INNI platform so far. Although MRI data had to meet some minimum requirements in order to be included,^12^ a full standardization of acquisition protocols was not requested from sites, at least in the first phase of the project.

The main challenges faced at the beginning of the INNI initiative were related to ethical approvals, to the creation of the online platform, to ensure proper handling of anonymity, and to define guidelines to regulate database access levels and their implementation as access procedures.^12^ Conversely, most of the subsequent challenges are related to the quality assessment (QA) of the data collected, which will now be used for different research projects at the 4 promoting sites. Systematic QA (on patient positioning, image inhomogeneity, distortions and artefacts, and measurement of contrast-to-noise ratio) has been established to verify source data and ensure maintenance of high quality. QA results will be used to propose effective guidelines on acquisition protocols and scanning options to improve harmonization of MRI data. Basic analysis (e.g., T2-hyperintense lesion segmentation, T1-hypointense lesion refilling, minimal preprocessing on diffusion-weighted MRI and resting-state fMRI scans) that may be shared in the INNI platform to harmonize future projects is also being performed in a centralized manner.

### Recommendations on MRI Data Sharing in MS

• Clearly define variables to be shared: this avoids ambiguity and heterogeneity at a later stage.
• Set up proper QA and quality control (QC) procedures to ensure compliance with minimum standards: preferably quantitative and automated, such procedures guarantee the integrity of the included data.
• Implement clear policies and procedures on how access to data can be obtained.
• Create a flexible data sharing system, permitting a manifold use of collected data: by choosing maximally permissive data licenses (within legal and institutional boundaries), combined with clear data storage organization, database management, and flexible access choices, data can be flexibly and easily selected, accessed, and used for a variety of purposes.

## Artificial Intelligence

### Artificial Intelligence in Medical Image Analysis Beyond MS

Artificial intelligence can be roughly divided into classical machine learning techniques such as support vector machines and (newer) deep learning techniques based on convolutional neural networks. Classical machine learning approaches typically make predictions using classifiers trained not directly on images, but on features extracted from images.^13^ While this can be advantageous, it precludes the discovery of features not perceptible to or appreciated by the human observer. Deep learning, when applied to classification or segmentation of images,^14^ instead analyzes image data directly, without prior feature selection. This has given rise to excellent classifier performance in a range of medical imaging applications.^13^ However, as shown for example by Ghafoorian et al.,^15^ at least given the current limitations regarding sizes of available datasets and networks, performance may be further improved by incorporating well-chosen features extracted from the images using domain knowledge and classical image analysis techniques. Specifically, in their work, they incorporate measures reflecting location in the brain to improve segmentation of age-related white matter (WM) hyperintensities.^15^

### Artificial Intelligence in Imaging of MS

Existing studies applying artificial intelligence in MS imaging can generally be divided into descriptive and predictive experiments. Descriptive studies use cross-sectional datasets in order to segment from MRI WM lesions^16^ or specifically contrast-enhancing lesions,^17^ identify imaging patterns based on MS phenotype or clinical or cognitive disease severity,^18^ or perform an automated diagnosis using uni- or multimodal information.^19^ Predictive studies, on the other hand, detect patterns in baseline data that allow for predicting future disease outcome or severity by incorporating clinical follow-up information.^20,21^ The majority of studies to date used classic machine learning techniques such as support vector machines or random forests, where features have to be defined and extracted from the data a priori, while more recent studies also use deep learning methods, allowing automated detection of relevant features in the data. Deep learning has now been used not only to segment WM lesions^22-24^ or their enhancing subset,^17^ but also to quantify lesion changes,^25,26^ detect the central vein sign,^27^ classify different lesion types based on diffusion basis spectrum imaging,^28^ predict gadolinium enhancement from other image types,^29^ perform MRI-based diagnosis,^30,31^ segment and analyze nonlesion structures,^32,33^ analyze myelin water fraction^34^ or quantitative susceptibility mapping data,^35^ synthesize absent image types,^36^ perform automatic QC,^37^ improve image quality,^38^ or correct intensity differences between scanners^39^ (additional references in eAppendix 1).

### Challenges of Artificial Intelligence in MS Imaging

Although showing impressive performance, state-of-the-art deep learning methods (convolutional neural networks) rely solely on the use of local intensity patterns and contextual features to guide the image analysis process. They lack high-level abstract thinking and have limited understanding of human anatomy and physiology. Learning from relatively small and noisy datasets, learning systems are commonly unable to extrapolate and handle uncertain situations. Furthermore, in precision medicine applications, fully automatic, robust, and quick measurements are required for every subject. Three important categories of current limitations of learning systems are inputs, labels, and uncertainty and confidence.

#### Inputs

The main limitation of many machine learning models is the strong dependence on (good) training data. While human raters may intuitively extrapolate from a few examples to new cases that may be very different, a supervised (deep) learning model has to be fed with sufficient examples to cover the whole range of heterogeneity in the population, disease, and scanning parameters. Differences in imaging devices, acquisition parameters, tissue contrasts, artefacts, and noise patterns can degrade algorithm performance if not handled appropriately. To overcome between-scanner or between-acquisition image differences, many approaches for postprocessing-based data harmonization have been proposed, including traveling phantoms,^40^ which requires physical travel of objects or subjects,^39^ limiting scalability; data augmentation,^41^ which requires sufficiently accurate signal simulating models; and domain adaptation,^42^ which has shown promising results. Ultimately, the most robust results may come from combinations of the above, together with basic steps such as intensity normalization (additional references in eAppendix 1)

Given the relatively low prevalence of MS, lack of training data is an issue of particular relevance here. Insufficient variability in training data can lead to overfitted models that do not perform well on new data, and single-center datasets seldom exceed a few hundred MS cases. This effect can be reduced with regularization, augmentation, and cross-validation, but not fully removed. Therefore, pooling data from different sources is advantageous, but this introduces new challenges due to differences between centers, scanners, and scanning protocols, which require standardizing and postprocessing.

The majority of published machine learning studies in MS used research data rather than clinical data, which has limitations: patients were filtered through inclusion criteria; the number of subjects and scans is limited by the obtained funding; and patients with more severe disease are more likely to drop out, biasing data towards more benign cases. Clinical data are more representative of the general (disease) population but are typically more heterogeneous and require additional patient consent.

#### Labels

Training with high-quality labels is crucial to attaining good performance of machine learning systems. Labels can be the person's diagnosis or other overall features, or typically in image analysis tasks, manual outlines of anatomical structures or pathologic entities like MS lesions. Distinguishing MS lesions in the WM from other WM lesions and from normal-appearing WM requires skill and expertise. The variability of labeling protocols and inter- and intrarater variability introduce errors when training machine learning systems. Such errors degrade the performance of learning systems and limit to what extent that performance can be validated. Those errors could be quantified by expanding training sets, based on common protocols applied by larger numbers of raters, and subsequently overcome by modeling or machine learning approaches.

#### Uncertainty and Confidence

Algorithms commonly solve a categorical hard problem, but clinical decisions are rarely categorical, and involve intrinsic uncertainty. The introduction of biomarker- and subject-specific error bars, and the development of novel ways to convey and introduce this information into the clinical workflow, will present challenges to clinical adoption. Recent work addresses that uncertainty for MS lesion detection and segmentation.^43^ Other areas of medicine with inherently uncertain predictions may suggest ways of introducing this uncertainty into the clinical workflow in the context of MS imaging (additional references in eAppendix 1).

### Recommendations on Machine Learning in MS Imaging

• Compile large, annotated datasets for training. To obtain sufficient amounts of training data, large-scale data sharing of MS imaging data is required, both for homogeneous datasets (for generating new knowledge), and heterogeneous datasets (for deriving more generalizable classifiers).
• Create methods that are robust to data variability. Harmonize data using both classical and machine learning techniques to improve robustness to unseen datasets.
• Include nonresearch data in training. Train machine learning methods also on data acquired in a real-life clinical setting, to increase robustness to heterogeneity and to improve applicability in the clinical population.
• Create high-quality labels. Validating algorithms for clinical use will require large multicenter labeling efforts yielding, depending on the aims, consensus-based “ground truth” labels or collections of individual raters' labels.
• Allow more subtle information in labels than global yes/no answers. The use of soft labels (e.g., image-wide disease classification) to model the intrinsic anatomical and pathologic variability should also be investigated.
• Incorporate the uncertainty of classifier predictions. Algorithms should learn the intrinsic uncertainty and confidence of every decision they make. Diagnostic and prognostic guidelines should be modified to enable clinical usage of biomarker- and subject-specific uncertainty metrics.

## Opportunity 1: Crowdsourcing

### Crowdsourcing in Research

Crowdsourcing, not to be confused with crowdfunding, refers to people donating their time and skills to complete certain tasks. In the context of scientific research, it is sometimes called “citizen science.” Its premise is that there are many enthusiastic members of the general public who are willing to donate some of their time to science. By making it easy for them to contribute, the scientific community can reward their enthusiasm and willingness by letting them help the field forward. Successful projects have been conducted this way, including examples in astronomy on differentiating different galaxy types (Galaxy Zoo^e70^), in organic chemistry on the topic of protein folding (Foldit, a game with tens of thousands of players^e71^), in biology on identifying bat calls (Bat Detective^e72^), and in paleontology on dinosaur limb bone measurements (Open Dinosaur Project^e73^). The potential for brain imaging applications has been noted, and a successful approach to interface-building, data management, and analysis has been described. SPINE,^e74^ Open Neuroimaging Laboratory,^e75^ and OpenNeuro^e76^ are examples of Web-based infrastructures for crowdsourced brain imaging research.

While the potential benefits of crowdsourcing to the researchers are clear, the benefits to the participants (the “crowd”) may be less obvious. There is the potential gratification of contributing to science, and in the case of MS research, these volunteers may be people with an interest in brain imaging or neuroscience, or they may know someone who has MS and want to help develop a solution. Furthermore, well-designed crowdsourcing activities can also carry the reward of being entertaining to perform. The field of “gamification” is a rapidly developing area of research and development in its own right that has already been applied in the radiologic field,^44^ which creates important opportunities for helping volunteers enjoy participating in crowdsourced research and remain committed to finishing their contribution.

### Potential for Crowdsourcing in MS Imaging

For processing large amounts of image data on a regular basis, as in a clinical setting, automation of analysis methods is key. The training that goes into such automated methods, whether based on deep learning or using other approaches, plays a large role in their performance. Ideally, reference labels, for example, of specific imaging features such as MS lesions or particular anatomical structures, would be generated by intensively trained expert raters. However, if this is not possible, for example due to the associated costs, crowdsourcing such training labels could provide a realistic alternative, if properly used. A potential issue concerns the quality of crowdsourced image annotations. For the example of image segmentation, this has been addressed. Specifically, Bogovic et al.^45^ demonstrated that for cerebellar parcellation, it is possible to achieve high-quality labels from a group of nonexpert raters.

This suggests that, provided that training and QA procedures are adequately employed, a large group of nonexpert volunteers could create reference labels on a large enough dataset to train deep learning or other methods robust to data variability. Nevertheless, as the task becomes more complex, the degree of communication required between the participants in order to achieve an adequate performance is likely to increase, with the risk of the efforts becoming an outsourcing initiative instead of a crowdsourcing one. Thus, the project to be carried out should be clearly defined in terms of tasks and expectations, with the inclusion of tutorials and support by an experienced professional in the field.

Besides providing training labels for image segmentation, crowdsourcing may also assist in other tasks such as (providing training labels for) image artifact detection, QC, or disease classification. Especially niche applications such as MS imaging, where the costs of expert training labels can be prohibitive, can provide a “sweet spot” where crowdsourcing can make a crucial contribution that advances the field. A first such approach has been proposed recently.^46^

### Recommendations for Crowdsourcing in MS Imaging

• Ensure high-quality instruction of volunteers. In order to help the crowd participate effectively, especially for longer and more complex tasks, comprehensive tutorials are essential.
• Define clear tasks and expectations. In order to allow volunteers to make contributions to research, their tasks should be clearly defined, and generally limited in scope and time investment.
• Enforce rigorous QC. In order to ensure high quality of the crowdsourced contributions, QC procedures such as repeatability and agreement with experts on selected samples are essential.

## Opportunity 2: Solutions to Personal Data Protection and Consent Requirements

### Personal Data Protection and Consent in the GDPR Framework

When sharing data, protecting personal data is a crucial guarantee to the participants. Because the MAGNIMS Study Group is a European collaboration, the expertise available on data protection laws in other jurisdictions is limited and we therefore focus here mainly on the situation in the European Union. Personal data protection is required by law in the European Union, with very specific rules set out in the General Data Protection Regulation (GDPR^e77^). While the specific legal requirements vary, concerns about preserving confidentiality of data of participants exist around the globe, and different legal frameworks to address these concerns exist in different countries. Differences between the GDPR and the US Health Insurance Portability and Accountability Act (HIPAA) framework include the more limited scope of the latter, and have been discussed in detail elsewhere.^47^ Personal data is understood as any information relating to an identified or identifiable natural person. An identifiable natural person is one who can be identified, either directly or indirectly. To determine whether someone is identifiable, all the means reasonably likely to be used have to be taken into account, and for each of these, the costs and the amount of time required for identification, the available technology at the time of the processing, and expected technological developments have to be assessed. This is important because GDPR holds the controller, that is, the researcher's organization, accountable. Security of personal data must be demonstrated through the existence of both technical and organizational measures.^e77^ GDPR places the persons whose data it concerns (referred to as “data subjects”) in full control of what happens to their data. If personal data are meant to be shared or the possibility of identification cannot be excluded, written informed consent must be obtained from all participants for data sharing, including whether data will be shared with countries where EU regulations do not apply. Furthermore, procedures must be in place for removal of data when participants ask for that removal. The data protection authorities consider coded or pseudonymized data as personal data. If personal data are to be shared, written data protection agreements are necessary to demonstrate compliance with the GDPR.

### Challenges Related to Personal Data Protection and Consent

Ensuring protection of personal data while providing adequate access for research purposes is challenging. Full anonymization, meaning that the person cannot be identified from the data at all, may be difficult to achieve in several cases. In “extreme” groups (e.g., rare diseases, extremely tall persons), some basic information accompanying the imaging data may help reveal the identity of the person. The increasing accumulation of big data on people's behavior from many different sources by many companies and organizations poses another hurdle to achieving full anonymization. New technologies, notably artificial intelligence techniques, allow, for example, reconstruction of faces from low-resolution pictures.^48^ In brain imaging, structural images allow 3D face reconstructions, possibly enabling identification. Face removal and face scrambling^49,50^ do not fully solve this, as these procedures may affect subsequent image analysis, for example, radiotherapeutic dose distributions or EEG signals, or further brain image analyses.^51^ Removed faces can be (partially) reconstructed, and the structure of each brain may soon be enough to identify the person.^52^

It may therefore be difficult to reach full anonymization at all. Hence, a second option may be more viable: to request, upfront, informed consent of the persons to share their data in an identifiable or not directly identifiable way. This informed consent should comply with national laws including those based on GDPR, where applicable, as well as indicate the various options for planned or as yet unforeseen data sharing, such as with researchers in countries outside the European Union, or perhaps with members of the general public through crowdsourcing initiatives as discussed above. In addition, to further ensure the protection of personal data, an agreement on the use of the personal data must be made between the institutions or organizations sharing the data. An increasingly important means of generating large cohorts is by sharing the data across large groups of many different centers from many countries, which can lead to additional legal uncertainties about personal data protection, data ownership, and data usage. The extreme case of this informed consent approach, where legally allowed, would be to ask the participants to consent to sharing their data without restricting that sharing to specific parties or applications.

A third way to share research data is by using an infrastructure that allows researchers to analyze the data remotely.^53^ Such “trusted data ecosystems” at their core are similar to a federated database but are much more comprehensive and encompass not just data management but all features necessary to perform analyses on the data including the computing infrastructure and audit trail. An example of such an infrastructure, currently under development in the Netherlands, is the Health-RI infrastructure.^e78^ The approach taken by Health-RI is that the personal data are on the inside and the platform performs the analyses, so researchers only receive the outcome measures but have no access to the actual data. Examples focused on federated deep learning, in which model parameters but not data are transferred between sites, are described by Chang et al.^54^ and Remedios et al.^55^ A limitation of such a federated approach is that the careful scrutiny of analysis pipeline success and inspection of intermediate results is less directly feasible than in the more standard data sharing approach, which would hamper not just any particular research project but also use of those data for further methodologic improvements. An advantage is that their comprehensive approach, including technological, legal, and business layers, ensures compliance with regulations, governance, legal issues for data sharing, security issues, and accountability.

The trade-off between protecting privacy and allowing access remains the main challenge that needs to be addressed properly. In this context, “differential privacy” may offer solutions. Limiting how much algorithms can learn from each data point prevents algorithms from learning enough to identify individuals, yet allows them to learn the relevant information at the population level.^56^

### Recommendations Related to Personal Data Protection and Consent

• Protect personal data. Implement technical, legal, and organizational guarantees for protecting personal data. For MRI, these include DICOM anonymization and face removal.
• Always request consent for data sharing. Maximize possibilities for reuse by requesting participant consent for subsequent sharing and aiming for a broad scope of future projects. Invest in standardization of the necessary data protection agreements.
• Invest in developing optimized infrastructure. Investigate how the strengths of Trusted Data Ecosystems can be combined with access to raw data and intermediate results.

## Opportunity 3: Organized Analysis Challenges

### Organized Analysis Challenges as a Tool for Accelerating Methodologic Developments

With image analysis and machine learning algorithms advancing at a rapid pace, there exists a need to understand the performance and limitations of state-of-the-art approaches. Evaluating the performance of an automated algorithm, such as lesion segmentation, is a fundamental part of methods development that can require significant resources. Grand challenges are organized, competitive events that provide data to be analyzed, an analysis objective, ground truth data, and evaluation metrics for achieving the objective.^e79^ They provide a means to compare the performance of multiple algorithms, to which a single laboratory would not typically have access. Furthermore, by providing these critical resources, research laboratories may participate in the challenge that might not otherwise engage in MS research.

The format of a grand challenge typically involves several key steps. Participating teams are first provided with a training dataset that includes both imaging data and the ground truth. For the aforementioned lesion segmentation challenges, this consisted of multicontrast MRI data from multiple patients and a set of manual lesion delineations. The training data allow the teams to optimize performance of their algorithms and achieve results consistent with the ground truth. Next, teams are provided with a test dataset that includes imaging data but no ground truth. The teams apply their approach to the test dataset and submit results for evaluation. Finally, the teams and organizers discuss performance of the different algorithms, as well as the evaluation and related issues.

There have been 3 segmentation challenges focused specifically on the segmentation of MS brain lesions: the 2008 MICCAI Challenge,^57,e106^ the 2015 ISBI Longitudinal Challenge,^58^ and the 2016 MICCAI Challenge.^59^ These provide clear examples of what can be achieved through this kind of approach. An enduring key benefit of these challenges, beyond the papers, is that the organizers have continued to make the data available after the meeting, and have set up Web-based systems for continually benchmarking new algorithms. In this way, all 3 challenges continue to actively make an impact, aiding software developers in developing improved methods.^e80-e82^

These advances notwithstanding, challenges thus far have only released portions of the full datasets for training, with the testing data reserved by the challenge organizers for algorithm evaluation. Furthermore, data use licenses have been restricted to research or educational use. Those previous MS challenges have also focused rather narrowly on different aspects of MS WM lesion segmentation. For example, the 2015 challenge focused on longitudinal data,^58^ while the 2016 data focused on multicentric data, including those acquired at different field strengths.^59^ Continued organization of challenges could target benchmarking algorithms for applications directly relevant to patient care, such as clinical trials or patient monitoring. Instead of metrics based on lesion segmentation accuracy, algorithms could be evaluated based on predicting the efficacy of therapies or clinical measures. Besides white matter lesion segmentation, a number of other promising imaging biomarkers could be tested: cortical lesions, cortical gray matter measurements, and thalamic volumes have all been found to be promising predictors of disease progression.^60^ An MS database with whole-brain labels, currently not available, would aid training and validation of algorithms to more accurately extract such biomarkers. Other grand challenges could examine MRI of spinal cord morphology and pathology and characterization of retinal morphology using optical coherence tomography. There is ample opportunity for challenges to contribute to further improvements in methods for studying MS, as well as proof from previous years that challenges can be a successful approach.

### Recommendations on Organized Analysis Challenges for MS Image Analysis

• Include additional aspects of MS image analysis other than WM lesions, such as cortical lesions and measures of brain volume.
• Evaluate algorithms also against clinical outcomes, instead of just against imaging data.
• Ensure challenge datasets contain large numbers of images and labels, to improve robustness and generalizability.
• Reduce restrictions on challenge data, to allow more diverse applications and to build more expansive data resources for algorithm development and evaluation.

## Discussion

To maximize improvements of both the understanding of MS disease processes and in vivo MRI methods to study those, using big data and machine learning, specific recommendations were provided on data sharing, machine learning, crowdsourcing, personal data protection, and organized analysis challenges.

## Glossary

GDPR: General Data Protection Regulation
INNI: Italian Neuroimaging Network Initiative
MS: multiple sclerosis
QA: quality assessment
QC: quality control
WM: white matter
